# Over Expression of Minichromosome Maintenance Genes is Clinically Correlated to Cervical Carcinogenesis

**DOI:** 10.1371/journal.pone.0069607

**Published:** 2013-07-17

**Authors:** Mitali Das, Shyam Babu Prasad, Suresh Singh Yadav, H. B. Govardhan, Lakshmi Kant Pandey, Sunita Singh, Satyajit Pradhan, Gopeshwar Narayan

**Affiliations:** 1 Cancer Genetics Laboratory, Department of Molecular and Human Genetics, Banaras Hindu University, Varanasi, Uttar Pradesh, India; 2 Department of Radiotherapy and Radiation Medicine, Banaras Hindu University, Varanasi, Uttar Pradesh, India; 3 Department of Obstetrics and Gynaecology, Banaras Hindu University, Varanasi, Uttar Pradesh, India; 4 Department of Zoology, Mahila Mahavidyalaya, Banaras Hindu University, Varanasi, Uttar Pradesh, India; University of Alabama at Birmingham, United States of America

## Abstract

Minichromosome Maintenance (MCM) proteins play important roles in cell cycle progression by mediating DNA replication initiation and elongation. Among 10 MCM homologues MCM 2–7 form a hexamer and assemble to the pre-replication complex acting as replication licensing factors. Binding and function of MCM2-7 to pre-replication complex is regulated by MCM10 mediated binding of RECQL4 with MCM2-7. The purpose of this study is to explore the role of MCMs in cervical cancer and their correlation with the clinical parameters of cervical cancer. We have investigated sixty primary cervical cancer tissue samples, eight cervical cancer cell lines and thirty hysterectomised normal cervical tissue. The expression profiling of MCMs was done using semi-quantitative RT-PCR, immunoblotting and immunohistochemistry. MCM2, 4, 5, 6, 7, 10 and RECQL4 are significantly over-expressed in cervical cancer. Among these, MCM4, 6 and 10 show increased frequency of over expression along with advancement of tumor stages. MCM4, 5 and 6 also show differential expression in different types of lesion, while MCM2 and MCM10 are over expressed in cervical cancer irrespective of clinico-pathological parameters. Our data indicates the role of MCM4, MCM5, MCM6, MCM10 and RECQL4 in the progression of cervical cancer.

## Introduction

DNA replication is the event of common interest in the study of initiation and progression of cancer. A normal cell maintains its entry and exit into cell cycle by several checkpoints and “licensing” its DNA replication only once per cell cycle. This licensing mechanism includes the formation of pre-replication complexes (pre-RCs) in late M and early G1 phases and their subsequent activation at the G1–S boundary. The pre-RCs mark the replication origins and control bidirectional DNA synthesis from these origins when S phase is initiated. Pre-RC assembly involves sequential recruitment of several proteins on replication origin. The reaction starts by the initial binding of origin recognition complex (ORC). Subsequent binding of CDC6 and CDT1 provide a landing pad for the further recruitment of putative DNA helicases as Minichromosome Maintenance (MCM) 2–7 complex [1]. Other important members of pre-RC are MCM10 and RECQL4 [2]. At the G1–S transition, the activity of two kinases, CDC7 and cyclins E/A-CDK2, recruit additional factors to pre-RCs, resulting in the formation of pre-initiation complexes (pre-ICs) [3]. Additionally, CDC7 and CDK2 activate the MCM2–7 helicases, which together with formation of pre-IC result in recruitment of DNA polymerases and initiation of DNA replication. Paradoxically, during late S and M phases, high activity of cyclin-dependent kinase (CDK) results in dissolution of the pre-RCs and destruction of selective pre-RC components, thereby preventing DNA re-replication [4].

MCM proteins were first recognized in the yeast *Saccharomyces cerevisiae* as mutants defective in the maintenance of mini chromosomes, suggesting a role in plasmid replication and cell cycle [1]. At least 10 homologues, MCM1-10, have been characterized in humans. Among these, MCM2-7 and MCM10 are involved in DNA replication [5]. Expression profiling of isolated MCM genes in multiple malignancies has been reported [6]. Deregulation of MCMs by reducing or increasing the levels of a single MCM leads to disruptions in genome stability in yeast [7], [8], [9]. Since MCM activity is essential for DNA replication in dividing cells and is lost in quiescence [10], MCMs are obvious markers for proliferation. Molecular studies suggest that increased levels of MCMs mark not only proliferative malignant cells [11], [12], but also precancerous cells and the potential for recurrence [13], [14]. Experimental evidence has identified RECQL4 and MCM10 as most important components of pre-RC [15]. During DNA replication, MCM10 mediates RECQL4 association with MCM2-7 complex on the origin [2]. RECQL4 is up regulated in actively proliferating virus transformed human B cells, fibroblasts and umbilical endothelial cells [16]. However, the expression profile of MCM10 with respect to RECQL4 and other MCMs is poorly understood in cancers. Since the expression level of MCM proteins in several dysplasias and neoplasias is up-regulated manifold, these proteins can be useful as potential diagnostic and prognostic marker for human malignancies [17].

Here, we show the expression profiles of MCM2, MCM3, MCM4, MCM5, MCM6, MCM7, MCM10 and RECQL4 in cervical cancer (CC) cell lines and primary tumors. We have also shown correlation of the expression levels to clinical parameters.

## Materials and Methods

### Primary Tumor Biopsies, Controls and Cell Lines

Sixty primary tumor biopsy samples, 30 hysterectomised controls and 8 cervical cancer cell lines have been used for this study. Tumor biopsies were collected in RNA later from the patients attending the Department of Radiotherapy and Radiation and normal cervical tissue were taken from subjects without any history of cervical cancer and who underwent hysterectomy for other reasons, in the Department of Obstetrics and Gynaecology, Institute of Medical Sciences, Banaras Hindu University, Varanasi. All biopsies were obtained after appropriate informed written consent of the subjects and approval of the institutional review board/ethical committee of the Institute of Medical Sciences, Banaras Hindu University. The biopsy samples were sub categorized according to the clinical information (Table 1). Eight cervical cancer cell lines (HeLa, SiHa, SW756, C-4I, C-33A, CaSki, MS751, and ME-180) [18] were kind gift from Dr. VVS Murty, Columbia University, New York, USA. HPV typing of primary tumor samples was done by PCR amplification using primers of HPV-16, HPV-18 and L1 consensus sequences [19].

**Table 1:** Patient characteristics.

parameters		Nos. of patients (%)
Age	≤40	14 (23.3%)
	41–50	22 (36.7%)
	51–61	18 (30%)
	>61	06 (10%)
Stage	I	06 (10%)
	II	22 (36.6%)
	III	32(53.4%)
Types of lesion	Infiltrative	08(13.3%)
	Ulceroinfiltrative	11 (18.3%)
	Proliferative	21 (35%)
	Ulceroproliferative	09 (15%)
	Ulcerative	11 (18.3%)
HPV types	HPV 16	43 (71.7%)
	HPV 18	01 (also HPV 16+ve)
	Other HPV types	17 (28.3%)

### PCR

Total RNA was isolated from tissue samples preserved in RNA later (Ambion, USA), using TriZol (Invitrogen, USA) and from cell lines harvested from semi confluent culture flasks. Good quality RNA (as confirmed by integrity of 28S and 18S rRNA on agarose gel and A_260_/A_280_ ratio) was reverse transcribed by cDNA kit (Applied Biosystems, USA) according to manufacturer’s protocol. The cDNA was used for PCR amplification by gene specific primers (Table 2). Primers were amplified in 2X PCR Master Mix (Thermo Scientific, USA) in a condition optimized through temperature and MgCl_2_ concentration gradient. All the PCR experiments were accompanied with no-template control and β-actin as internal control. After agarose gel electrophoresis, band intensities were calculated by *SpotDenso* densitometry software (AlphaImager, USA) and normalized with β-actin. Thus a normalized expression level indicated the ratio between band intensity of the target gene and β-actin for each sample. A gene was considered to be up-regulated when it had a normalized band intensity value higher than *mean ±2× standard deviation* (SD) of normal cervix. Fold change for each sample is calculated by the expression value of a particular sample divided by average of the normal for a particular gene.

**Table 2:** Primer sequences.

Gene	Sequence (5′–3′)
MCM2F	AAT TTC GTC CTG GGT CCT TT
MCM2R	CAC TTT GCC TGG ACT CTC CT
MCM3F	CTT TCC CTC CAG CTC TGT CTA
MCM3R	TCA CCA GGC TTC GCT TTA TC
MCM4F	TTC TTT GAC CGT TAC CCT GA
MCM4R	ACA CTT GGC ACT GGA AGA AG
MCM5F	TAT TGC CTA CTG CCG AGT GA
MCM5R	ACT GTC CCT CTC GTG CTG AC
MCM6F	AAG CAC GTG GAG GAG TTC AG
MCM6R	CGC ACG TCC ATC TTA TCA AA
MCM7F	CCA GTC TCC CAC TTT CAT GC
MCM7R	CCA TCA CAG GGA ATG AAT GT
MCM10F	CCG TCT GCA AAA ATC CCC TGA GA
MCM10R	ATG AGC TTT TGG GAT CTG GAG GTG
RECQL4F	TCA TGG ATG ACC AGG TGT CT
RECQL4R	CTC ATC AAT GCA GGC AAA AG

### Immunoblotting

Whole protein fraction was isolated from cell lines (n = 8) primary tumors (n = 6) and control samples (n = 6) using lysis buffer (50 mM Tris-Cl _P_H7.4, 1 m EDTA, 2 mM EGTA, 1 mM DTT, 100 mM PMSF, 100 mM NaCl, 1% NP-40, 0.1% SDS and protease inhibitor cocktail). Protein concentration was determined by Bradford assay. 100 µg of protein per sample was run on SDS-PAGE and transferred on PVDF membrane (Millipore, USA). Mouse monoclonal anti-MCM4 antibody (Santa Cruz Biotechnology, USA) was used and the blots were detected with NBT-BCIP substrate system against ALP-tagged secondary antibody.

### Immunohistochemistry

Immunohistochemistry was done on paraffin embedded tissue sections. 5 µm thick sections of primary tumor (n = 2) and control (n = 2) were cut and a microarray of two subsequent sections of each sample were mounted on poly L- lysine coated slides. Tissue sections were deparaffinized and treated for 10 minutes in citrate buffer for antigen retrieval in water bath at 95°C. Rabbit polyclonal anti-MCM4 primary antibody (Abcam, UK) and rabbit polyclonal anti-Ki67 antibody (Pierce Biotechnology, USA) were used in 1∶250 and 1∶1000 dilutions respectively. Peroxidase conjugated secondary antibody was used against the primary antibody. For chromogenic detection, 3, 3′-diaminobenzidine tetrahydrochloride (DAB) (Sigma, USA) was used as the substrate for peroxidase. Slides were counterstained with hematoxylin. Cells with brown nuclei were considered as positively stained for MCM4. Negative control experiment was performed without using primary antibody.

### Statistical Analysis

Statistical analysis was done by SPSS version 16.0 for windows. Mann-Whitney and Kruskal-Wallis tests were used to compare two or more than two groups respectively. Where a Kruskal-Wallis test gave significant result, two individual groups of that particular parameter were compared by Mann-Whitney test. Expression trends among different stages were analyzed by Spearman correlation as required. Data is represented in the text as (mean of expression level ± SD) for expression level. P values <0.05 were considered as significant.

## Results

### Expression Profiling of MCMs and RECQL4 in CC Cell Lines and Primary Tumors

Our semi-quantitative RT-PCR analysis of MCM2-7, MCM10 and RECQL4 in primary tumors and CC cell lines reveals that MCM2, MCM4, MCM5, MCM6, MCM10 and RECQL4 are frequently up-regulated in primary tumors and CC cell lines, while MCM3 does not show significant change. MCM7 shows significant up regulation in primary tumors but not in CC cell lines.

**Figure 1:**
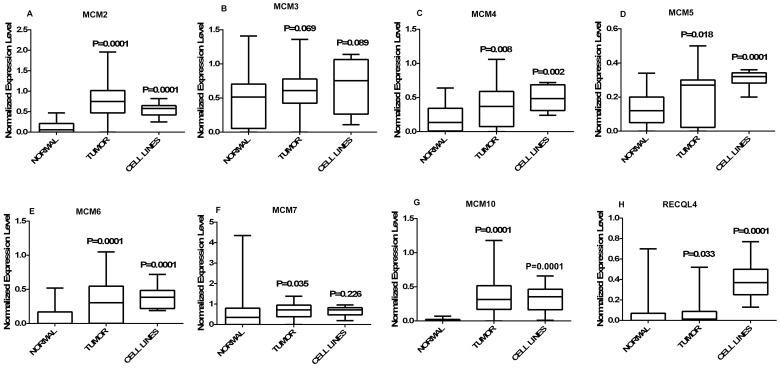
Graphical representation of normalized expression values. (A–H) Box plots representing the distribution of normalized expression values of normal (n = 30), tumor (n = 60) and CC cell lines (n = 8). A box in a given box plot represents the interquartile range (25^th^ percentile to 75^th^ percentile), the middle line denotes median and the extreme ends of the whiskers marks the minimum and maximum values. P-values indicated over each box represent the asymptotic significance (2-tailed) of Mann-Whitney test comparing normal to tumor and normal to cell lines independently.

**Figure 2:**
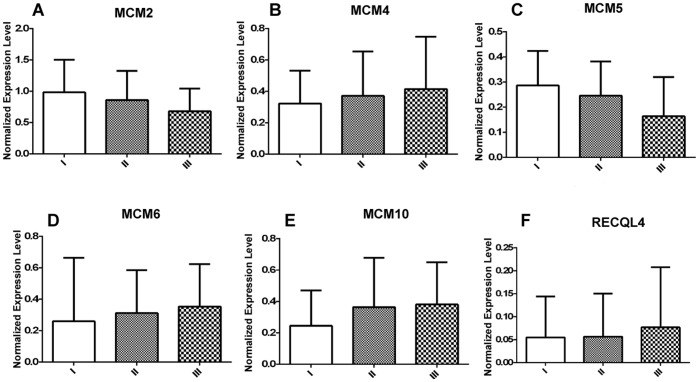
Expression of MCM genes in different tumor stages. Normalized expression level of (A) MCM2, (B) MCM4, (C) MCM5, (D) MCM6, (E) MCM10 and (F) RECQL4 in different tumor stages. I, II, III denote Stage I (n = 06), Stage II (N = 22) and Stage III (n = 32) respectively. Each axis represents mean ± SD.

**Figure 3:**
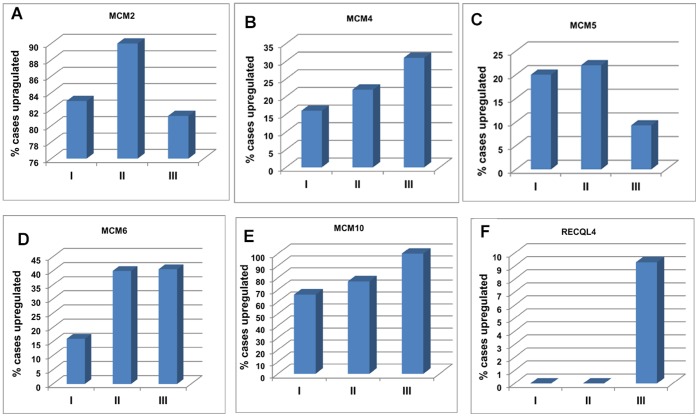
Frequency of over expression of MCM genes with advancing stage of disease. Percentage of cases showing over expression of (A) MCM2, (B) MCM4, (C) MCM5, (D) MCM6, (E) MCM10 and (F) RECQL4 in each tumor stage. I, II, III denote Stage I (n = 06), Stage II (N = 22) and Stage III (n = 32) respectively.

MCM2 shows significant up regulation (p = 0.0001) in primary tumor (n = 60, 0.777±0.425, median = 0.75, p = 0.0001) and cell lines (n = 8, 0.552±0.176, median = 0.58, p = 0.0001) compared to normal (n = 30, 0.118±0.138, median = 0.06) (Figure 1A). The average fold change of MCM2 in primary tumors is 6.56 (±3.6) and in cell lines is 4.6 (±1.48). The average expression level of MCM2 (Figure 2A) does not show any significant difference in different tumor stages. 83% cases of stage I, 90% cases of stage II and 81.2% cases of stage III show up regulation of MCM2 (Figure 3A). The Spearman correlation test does not show any significant correlation between expression level and increasing tumor stages (Spearman’s rho = −0.229, p = 0.078). MCM2 is over expressed irrespective of tumor lesion types (data not shown).

MCM3 do not show significant change in the expression levels of tumor (0.603±0.388, median = 0.61, p = 0.069) and cell lines (0.705±0.398, median = 0.75, p = 0.089) compared to normal (0.49±0.39, median = 0.5) (Figure 1B).

**Figure 4:**
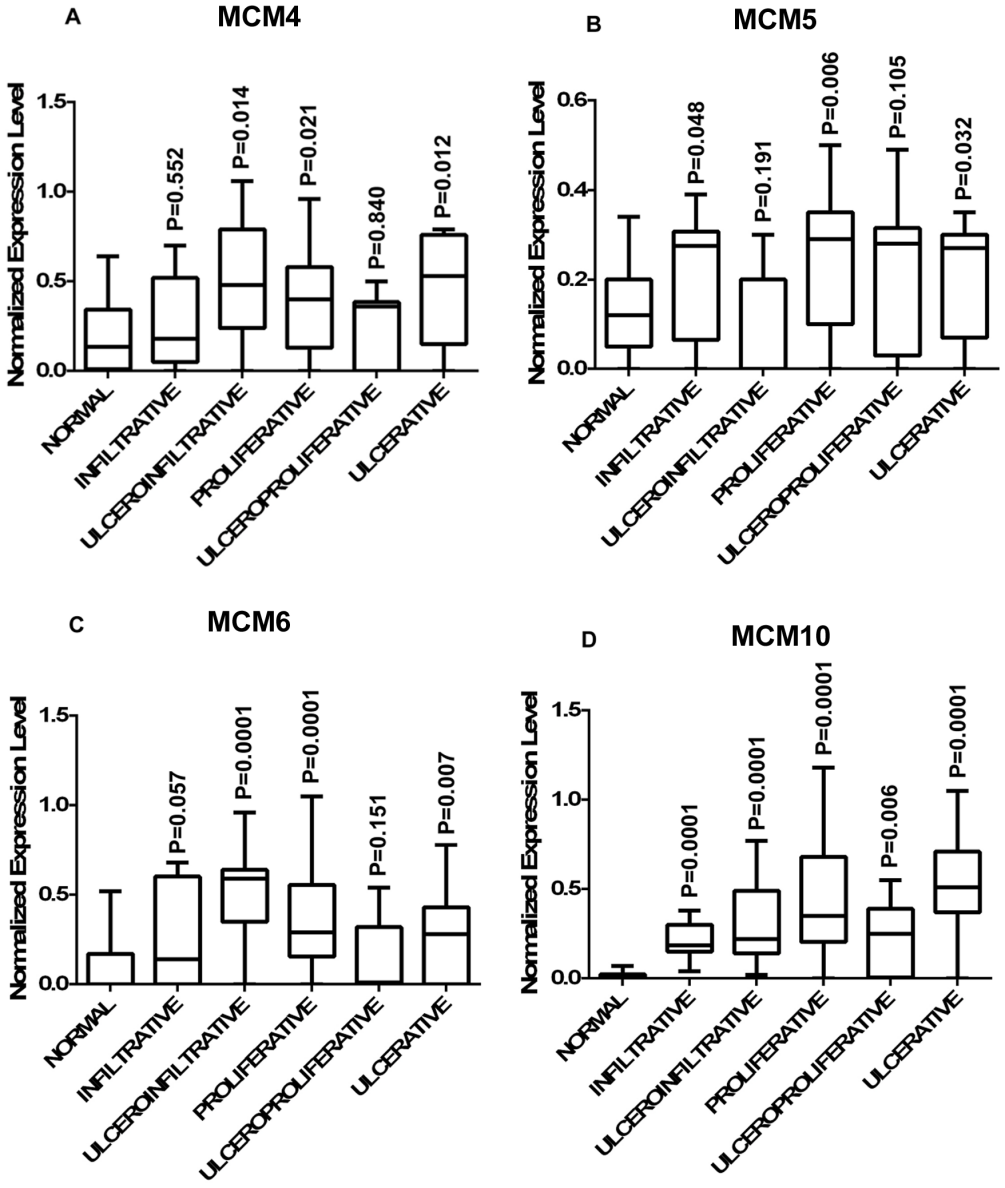
Distribution of expression of MCMs in different types of lesion of cervical cancer. Differential expression of (A) MCM4; (B) MCM5; (C) MCM6 in different types of lesions viz. infiltrative (n = 08), ulceroinfiltrative (n = 11), proliferative (n = 21), ulceroproliferative (n = 09), proliferative (n = 11) as compared to normal; (D). MCM10 show significant up regulation compared to normal in all different types of lesions. A box in a given box plot represents the interquartile range (25^th^ percentile to 75^th^ percentile), the middle line denotes median and the extreme ends of the whiskers marks the minimum and maximum values. P-values indicated over each box represent the asymptotic significance (2-tailed) of Mann-Whitney test comparing normal to a particular group.

**Figure 5:**
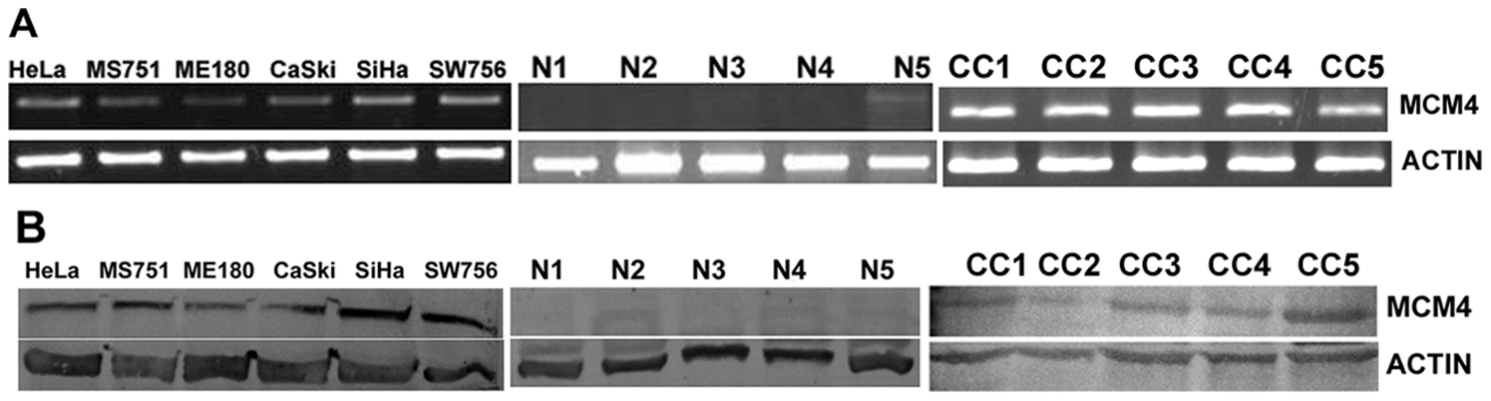
MCM4 expression. (A) Electrophoregram of semi-quantitative RT-PCR showing MCM4 expression in cell lines, primary tumors (CC1–CC5) and normals (N1-5). (B) Immunodetection of MCM4: Expression of MCM4 protein in cell lines; normals (N1-5) and tumors (CC1-5).

**Figure 6:**
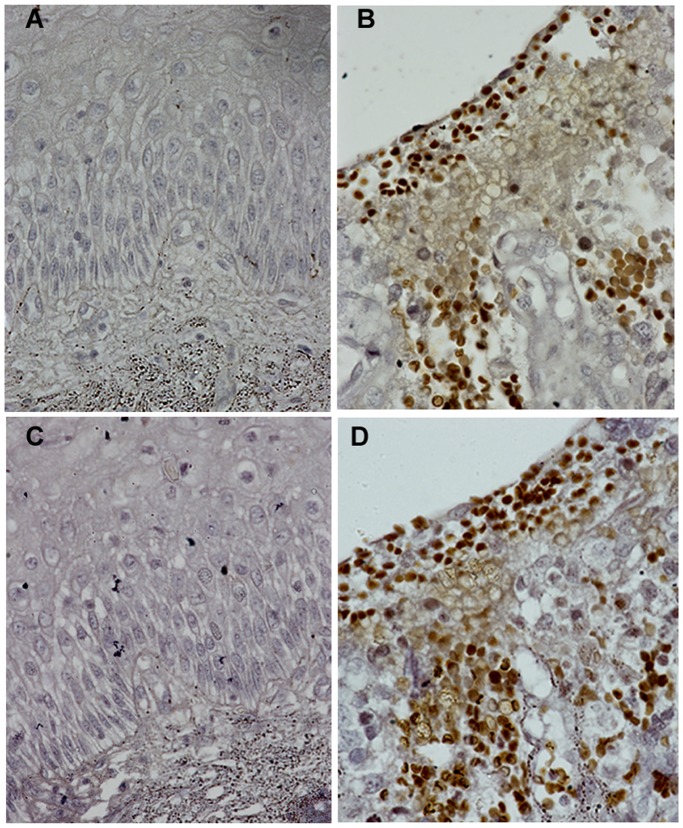
Immunohistochemistry. Immunohistochemical analysis of MCM4 expression in (A) Normal cervix; (B) Cancerous cervix; Ki67 expression in (C) Normal cervix; (D) Cancerous cervix (60X).

MCM4 is significantly over expressed in primary tumors (0.389±0.302, median = 0.37, p = 0.008) as well as in cell lines (0.485±0.193, median = 0.135, p = 0.002) as compared to normal (0.185±0.185, median = 0.135) (Figure 1C). The average expression level of MCM4 gradually increases with the advancement of the disease (Figure 2B). Frequency of MCM4 over-expression gradually increases with the advancement of stage of disease. It is over expressed in 16% cases of stage I (n = 06), 22% cases of stage II (n = 22) and 31% cases of stage III (n = 32) of the disease (Figure 3B). There is a trend of increasing MCM4 expression with the advancement of the disease, however, correlation is not significant (Spearman’s rho = 0.079; p = 0.546). Kruskal-Wallis test comparing expression levels of normal and different types of lesion shows significance (p = 0.026). Interestingly not all the groups, but ulceroinfiltrative (p = 0.014), proliferative (p = 0.021) and ulcerative (0.012) show significant difference compared to normal (Figure 4A). In primary tumors and cell lines, average fold changes in MCM4 expression is 2.08 (±1.6) and 2.61 (±1.04) respectively. Immunoblotting data is concordant with the RT-PCR data for MCM4 showing up-regulation in tumors (Figure 5). Immunohistochemical analysis shows that MCM4 expression is undetectable in the normal cervical epithelium (Figure 6A) whereas the MCM4 positive cells are dispersed in the cancerous cervical epithelium (Figure 6B). Proliferation in the cancerous cervical epithelium is confirmed by the expression of Ki67 in comparison to normal (Figure 6C and Figure 6D).

MCM5 shows significant up-regulation in primary tumor (0.206±0.152, median = 0.27, p = 0.018) and cell lines (0.305±0.05, median = 0.32, p = 0.0001) compared to normal (0.128±0.098, median = 0.12) (Figure 1D). Average expression level of MCM5 has been shown in figure 2C. 20% cases of stage I, 22% cases of stage II and only 9.3% cases of stage III show up regulation of MCM5 (Figure 3C). No positive correlation is found between expression levels of MCM5 with increasing tumor stages (Spearman’s rho = −0.308, p = 0.078). Kruskal-Wallis test shows significant difference between the expression level of MCM5 in normal and different types of lesions (p = 0.007). There is no difference found among the groups but individually some of the groups show differential expression compared to normal. MCM5 shows significant up regulation in infiltrative (p = 0.048), proliferative (p = 0.006) and ulcerative (p = 0.032) types of lesions as compared to normal (Figure 4B).

MCM6, which we found to be up-regulated in tumor (0.328±0.283, median = 0.305, p = 0.0001) and in cell lines (0.387±0.178, median = 0.385, p = 0.0001) compared to normal (0.082±0.145, median = 0) (Figure 1E). MCM6 shows an average of 3.9 (±3.4) fold increase in primary tumors and 4.6 (±2.15) fold increase in cell lines. Average expression level of MCM6 increases with the increasing tumor stages (Figure 2D). Frequency of MCM6 over expression increases with advancement of the disease as, this gene is over expressed in 16% cases of stage I, 40% cases of stage II, and 40.6% cases of stage III of the tumor (Figure 3C). Spearman correlation indicates a positive correlation between expression level and tumor stages (Spearman’s rho = 0.148, p = .259). Similar to MCM4, MCM6 also shows differential expression in different lesion types. MCM6 expression is significantly different in ulceroinfiltrative (p = 0.0001), proliferative (p = 0.0001) and ulcerative (p = 0.007) as compared to normal (Figure 4C).

MCM7 shows significant difference between tumor (0.674±0.396, median = 0.72, p = 0.035) and normal (0.574±0.844, median = 0.35), but cell lines (0.65±0.245, median = 0.715) do not show significant difference (Figure 1F). The average fold change of MCM7 in tumor is 1.15(±0.68) and in cell lines 1.11(±0.414). MCM7 expression does not show significant correlation with tumor stages (Spearman’s rho = −0.087, p = 0.557) or any difference in different types of lesion in comparison to normal (data not shown).

Expression level of MCM10 in tumor (0.367±0.277, median = 0.315) and cell lines (0.325±0.205, median = 0.35) is significantly different (p = 0.0001) from that of normal (0.016±0.022, median = 0) (Figure 1G). Maximum change was observed in the expression levels of MCM10 i.e. an average of 22.9 (±17.33) fold change in tumor and 20.14 (±12.7) fold change in cell lines. Mean expression level of MCM10 increases according to the increasing tumor stages (Figure 2E). 66% cases of stage I, 77% cases of stage II and 100% cases of stage III show MCM10 over expression (Figure 3E). Although, there is an increasing trend, no significant correlation (Spearman’s rho = 0.108, p = .410) is found between expression level and tumor stages. Also, MCM10 is significantly up-regulated in all lesion types compared to normal (Figure 4D).

RECQL4 expression is significantly different in tumor (0.067±0.113, median = 0.014, p = 0.033) and in cell lines (0.396±0.199, median = 0.37, p = 0.0001) than normal (0.059±0.154, median = 0) (Figure 1H). RECQL4 expression is limited up to an average of 1.12(±1.9) fold change in tumor but in cell lines it goes up to 6.7(±3.3) folds in cell lines. Average expression level and frequency of occurrence in each tumor stage has been shown in Figure 2F and Figure 3F respectively. No significant correlation (Spearman’s rho = 0.077, p = 0.557) is indicated between expression level and tumor stages, however, there is a positive trend.

### HPV Status

All the primary tumors were HPV positive, among which more than 71% were positive for HPV 16. Of eight cell lines C-4I, SiHa, were HPV 16 positive, HeLa, Me-180, MS751 and SW756 were HPV 18 positive and CaSki was both HPV 16 and 18 positive, while one cell line (C33A) was HPV negative. There was no significant correlation of the expression of any of the MCMs with HPV status or with HPV types.

## Discussion

Replication licensing can be positively correlated with the proliferative potential of eukaryotic cells [20]. Perpetually growing tumor cells require continuous licensing. Many tumors such as osteosarcoma [21], ductal breast carcinoma [22], medulloblastoma [23], prostate carcinoma [24], oral squamous cell carcinoma [25] and many others show over-expression of minichromosome maintenance genes. There are isolated reports of the deregulated expression of individual MCMs in cervical cancer. A recent immunohistochemical study has shown that MCM2 is differentially expressed in normal epithelium compared to high grade cervical intraepithelial neoplasia (CIN) and invasive cancer [26]. MCM3 and MCM4 have been shown to be over-expressed in CC cell lines [27]. Unlike the previous study, MCM3 does not show any significant change in our analysis, while MCM4 is significantly up-regulated. Western blot analysis and immunohistochemistry results are concordant with RT-PCR expression profiles. Since the expression of this helicase is relatively more frequent in higher grade tumors, it may serve as potential stage-specific marker for CC.

Dosage alterations of these replication associated genes have vivid cytogenetic background. MCM2 which is over-expressed in cervical cancer irrespective of any clinical parameter is located at 3q21. 3q21 shows high level of amplification in seven CC cell lines [28]. Overall, 3q shows frequent copy number gains by comparative genomic hybridization in cervical cancer [29]. Comprehensive cytogenetic approaches marked 8q as a region of high chromosomal gain in CC cell lines [30]. Two of the replisome associated genes, MCM4 (8q11.2) and RECQL4 (8q24.3) are included in this region. MCM4 has been detected as osteosarcoma driver gene as found to be over-represented in both copy number and expression profiles [21].

Not all but some of the genes as MCM4, MCM6, MCM10 and RECQL4 show a positive trend with increasing tumor stages. The discrepancy of correlation of MCM2, MCM5 and MCM7 may be due to a very small sample size in tumor stage I (N = 06). However, the differential over-expression of MCM4 and MCM6 in our study indicates their critical role in CC progression. Frequent over expression of MCM10, which partners with RECQL4 and binds to MCM2-7 complex activating its helicase activity, indicates that MCM10 is critical in the tumor progression.

In conclusion our study provides a comprehensive report of the expression profile of all the major MCM genes involved in human DNA replication and RECQL4, an important replisome associated factor in cervical cancer. Studies with larger sample size specifically of lower tumor stages can show significant correlation between expression levels of these genes and progressing tumor stages. This may give a better idea about the potentiality of these genes as stage specific markers. Further studies with precancerous lesions may provide clues as to whether these MCMs and RECQL4 can be therapeutic targets in cervical cancer.
